# J-shaped relationship between stress hyperglycemia ratio and delirium risk in critically ill patients: A population-based study

**DOI:** 10.1371/journal.pone.0350652

**Published:** 2026-06-05

**Authors:** Yingyang Li, Mengyuan Qiao, Hui Yang, Lu Chen, Haiyan Wang

**Affiliations:** 1 The First Affiliated Hospital, and College of Clinical Medicine of Henan University of Science and Technology, Luoyang, China; 2 School of Nursing, Henan University of Science and Technology, Luoyang, China; 3 People’s Hospital of Xinjiang Uygur Autonomous Region, Urumqi, China; Pelita Harapan University Faculty of Medicine: Universitas Pelita Harapan Fakultas Kedokteran, INDONESIA

## Abstract

**Background:**

The incidence of delirium in critically ill patients is strongly correlated with poor prognosis. The stress hyperglycemic ratio has emerged as a novel marker for assessing the response to acute hyperglycemia. Glycemic fluctuations during periods of stress play a crucial role in precipitating or directly causing delirium. However, the association between SHR and delirium in hospitalized ICU patients remains uncertain.

**Objective:**

This study aimed to investigate the potential relationship between SHR and delirium in ICU patie nts and to examine possible subgroup differences in this association.

**Methods:**

A total of 2,093 Intensive care unit (ICU) patients were included in this retrospective cohort study. The relationship between SHR and delirium was explored using multifactorial logistic regression, subgroup analyses, smoothed curve fitting, and threshold effect analysis models.

**Results:**

Among the 2,093 participants, 59.05% were male and 40.95% were female, with a mean age of 64.19 ± 16.31 years. We identified a non-linear positive correlation between SHR and delirium, with an inflection point at 0.68, and the odds ratio (95% CI) after the inflection point was 1.88 (1.35, 2.62), *P* < 0.001. This interaction was statistically significant concerning the APACHE II scores and C-reactive protein levels at admission.

**Conclusion:**

We found a nonlinear positive association between SHR and delirium. Our study highlights that managing SHR levels in critically ill patients may help to prevent or mitigate the development of delirium, emphasizing the potential value of SHR as an early intervention and precision treatment for delirium.

## Introduction

Delirium is an acute and sudden brain dysfunction characterized by fluctuations in consciousness, cognitive changes, and inattention [1]. Patients in the Intensive Care Unit (ICU) are at high risk for delirium, with an incidence ranging from 4% to 55% [2]. When delirium occurs in ICU patients, it may be accompanied by cognitive decline and neuronal damage [3], which in turn prolongs the length of hospital stay and causes higher mortality [4]. Additionally, delirium is associated with adverse nursing events, such as unplanned extubation and falls. The financial burden of delirium in ICU patients is substantial, with healthcare expenditures reaching $164 billion annually in the United States [5], while in Europe it is as high as $182 billion [6]. Evidence suggests that up to 60% of delirium is unrecognized in clinical settings [7], and early recognition and prevention of delirium is a significant challenge for healthcare professionals.

Regarding the pathogenesis of delirium, it is still unclear so far, and there are several main theories [8–10], including neurotransmitter alterations, stress response, sleep-wake cycle disorders, inflammatory response, electrolyte imbalance and genetic factors. Nevertheless, it is evident that delirium constitutes not merely a mental alteration but a clinical syndrome rooted in pathophysiological changes. Evidence suggests that fluctuations in blood glucose levels during periods of stress are thought to play an important role in inducing or directly causing delirium [11,12], which may be related to the fact that high blood glucose in stressful situations induces oxidative stress, disrupts the blood-brain barrier, and leads to subsequent neuronal damage [13,14].

The temporary rise in blood glucose observed in critically ill patients during acute stress, such as major trauma and sepsis, resulting from disturbances in substance and energy metabolism, is referred to as stress hyperglycemia (SHG). This acute response is mediated by the hypothalami-pituitary-adrenal axis and the sympathetic nervous system, leading to impaired neutrophil function, hyperosmolar diuresis, cerebral hypoxia, increased susceptibility to infections, and challenges in infection control, ultimately affecting disease prognosis [15,16]. The Stress Hyperglycemia Ratio (SHR) has emerged as a valuable biomarker, proposed in recent years to accurately identify and quantify the extent of stress hyperglycemia by calculating admission glucose and HbA1c levels, while adjusting for the patient’s chronic glycemic status over the preceding two or three months [17]. Although SHR provides valuable insights for diagnosing and predicting the risk of various conditions, its relationship with delirium has not been extensively explored. Therefore, determining whether SHR has potential value in predicting the incidence of delirium in ICU patients was the primary objective of this study. Thus, the primary goal of this study was to ascertain whether SHR could serve as a predictive tool for delirium incidence in ICU patients.

## Methods

### Study population

This retrospective observational cohort study was conducted at a comprehensive hospital in the Xinjiang Uygur Autonomous Region. On 15 January 2024, we conducted a retrospective analysis of clinical data from 2,093 patients admitted between 1 January 2021 and 31 December 2023, obtained from the electronic medical record system of this hospital. Patients without a history of diabetes who were at least 18 years old were included in the trial. Patients were excluded based on the following criteria: 1) long-term use of medications impacting glucose levels; 2) persistent coma, defined by a Richmond Agitation Sedation Scale score of −4 or −5 throughout their ICU stay; 3) diagnosis of diabetic ketoacidosis or hyperosmolar hyperglycemic state; 4) admission due to alcohol intoxication or who received a blood transfusion; 5) presence of an autoimmune disease at the time of admission; 6) incomplete data. The selection process is detailed in S1 Fig.

All methods were carried out in accordance with relevant guidelines and regulations. We followed the STROBE statement in reporting this study [18]. The study was approved by the hospital ethics committee (**approval number: KY2023071303**), which waived the requirement for informed consent because the medical records were de-identified and the study was retrospective.

### Delirium definition

Delirium was assessed using the confusion assessment method for the ICU (CAM – ICU) that has good validity and reliability. The CAM – ICU can be used in patients who are unable to speak, such as those in mechanical ventilation. A simplified Chinese version of the CAM – ICU has been affirmed for ICU patients. Delirium assessment was performed twice daily (between 10 AM and 6 PM) until the patient was discharged from the ICU. There are four main features of CAM – ICU: (1) acute onset or fluctuating course; (2) inattention; (3) altered level of consciousness; and (4) disorganized thinking.

The CAM – ICU was positive when features (1) and (2) with either (3) or (4) were present (https://www.icu delirium.org/resource-downloads/cam-icu-training-manual). We assessed patients in delirium as they had a positive CAM,ICU at any time during the entire ICU stay. The Richmond Agitation Sedation Scale (RASS) was used to assess the depth of sedation and agitation. It consists of a 10-point sedation scale (ranging from 5 to +4). The RASS was used firstly to assess the level of consciousness; coma was defined as the RASS score was 4 or 5. When the RASS score ranged from +4–3, the CAM,ICU was used for further assessment.

### SHR definition

HbA1c-adjusted glycemic variables were calculated as follows [17]: (1) A1c-derived average glucose (ADAG) levels: 1.59 × HbA1c (%) −2.59; (2) stress hyperglycemia ratio: [glucose/ADAG].

### Other covariates

The covariates in this study were based on previous reports in the literature and were associated with delirium and SHR. The clinical data of all patients were collected retrospectively by searching our clinical electronic database. The following covariates were included: (1) demographic indicators such as age (years), sex (male, female); (2) the anthropometric indicator body mass index (BMI), which was calculated by dividing weight (kilograms) by the square of height (meters); (3) history of disease included hypertension, congestive heart failure, cerebrovascular diseases, chronic liver disease, chronic kidney disease, chronic pulmonary disease; (4) disease-related factors including mechanical ventilation, APACHE Ⅱ points on the first day in ICU, length of ICU stay and hospital mortality. The study also encompasses laboratory findings on the first day in ICU, including haemoglobin (Hb), creatinine, albumin (Alb), and C-reactive protein (CRP). All blood samples were obtained from the patients on the second day of admission during the early morning of fasting.

### Statistical analysis

Continuous variables conforming to a normal distribution are denoted by (x ± s), and one-way ANOVA was used for comparison between multiple groups; continuous variables not conforming to normal distribution was expressed as *M* (*P*_*25*_*, P*_*75*_)*,* and the non-parametric Kruskal-Wallis H test was used for comparison between multiple groups; and categorical variables was described using frequency and constitutive ratios (%), and chi-square test was used for comparison between groups.

We calculated variance inflation factors (VIF) for all covariates (S1 Table). Spearman correlation was used to assess the relationship between delirium and SHR (S2 Table). A multivariate logistic regression model was used to examine the relationship between SHR and delirium in this cross-sectional analysis. To assess the effect of SHR on endpoints, ratio of ratios (OR) and 95% confidence intervals (CI) were calculated for quantification. To verify the robustness of our results across groups, subgroup analyses of age, BMI, cerebrovascular diseases, APACHE Ⅱ value, hemoglobin, C-reactive protein, length of ICU stay, and mortality were used. To assess dose-response relationships, we utilized restricted cubic spline (*RCS*) analysis, with additional two,stage comparisons based on the turning points of the RCS curves. In this study, we employed a weighted approach to reduce data volatility [19].

All statistical analyses were performed using R (version 4.4.1). Two tailed *P* values <0.05 were considered statistically significant. Detailed statistical analyses were provided in the supplementary material.

## Results

### Baseline characteristics

**Fig 1:**
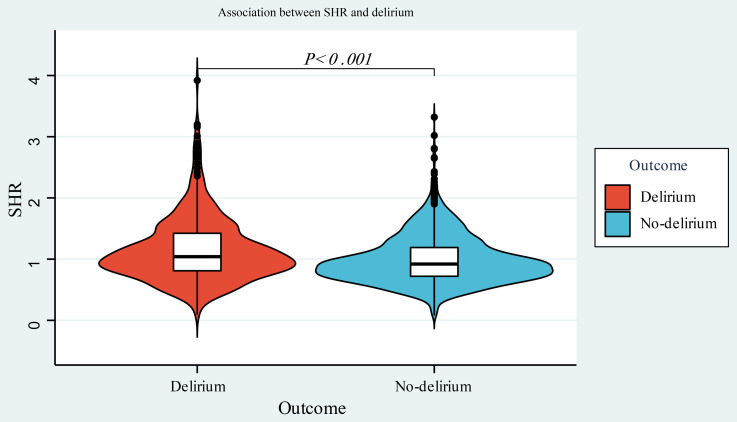
Violin plot of the relationship between SHR and delirium distribution.

Based on the inclusion and exclusion criteria, a total of 2,093 adult participants admitted to the ICU were included in the study. This population comprised 59.05% males and 40.95% females, with a mean age of (64.19 ± 16.31) years. The use of violin plots to demonstrate the relationship between SHR ratios and delirium showed that patients who developed delirium had a more centralized distribution of SHR ratios and higher scores (Fig 1). The histogram is used to display the SHR distribution (S2 Fig**).**

We classified the participants into two groups: those who did not had delirium and those who did, based on their experiences during the ICU stay. Subjects who developed delirium were likely to be of advanced age, high BMI level, history of cerebrovascular disease, low albumin, high APACHE II score on admission, and prolonged hospitalization (Table 1). In addition, we found that patients with episodes of delirium had higher SHR levels than those without delirium. There was a trend for SHR levels to be higher in patients with advanced age, high BMI levels, a history of cerebrovascular disease, high APACHE II scores on admission, and those who experienced delirium (Table 2).

**Table 1:** Characteristics of participants enrolled in the study by delirium.

Characteristics	Total (*n* = 2093)	No delirium (*n* = 1649)	Delirium (*n* = 444)	*P-value*
Age (years), median (IQR)	66.00(54.00,77.00)	65.00(53.00,76.00)	70.00(57.00,80.00)	<0.001^a^
Male, *n* (%)	1236(59.05%)	976(59.19%)	260(58.56%)	0.811^b^
BMI (kg/m^2^), median (IQR)	23.90(20.35,26.80)	22.70(20.10,26.50)	25.85(21.10,28.00)	<0.001^*^
Smoking, *n* (%)	548(26.18%)	442(26.80%)	106(23.87%)	0.213^b^
Drinking, *n* (%)	322(15.38%)	262(15.89%)	60(13.51%)	0.218^b^
Mechanical ventilation, *n* (%)	589(28.14%)	449(27.23%)	140(31.53%)	0.074^b^
APACHE Ⅱ points on the first day in ICU (points), median (IQR)	22.00(19.00,25.00)	22.00(18.00,24.00)	21.00(20.00,26.00)	0.007^*^
SHR, median (IQR)	0.94(0.73,1.24)	0.92(0.72,1.19)	1.04(0.80,1.43)	<0.001^*^
**Comorbidity**				
Hypertension, *n* (%)	811(38.75%)	640(38.81%)	171(38.51%)	0.909^b^
Coronary heart disease, *n* (%)	519(24.80%)	402(24.38%)	117(26.35%)	0.393^b^
Cerebrovascular disease, *n* (%)	476(22.74%)	348(21.10%)	128(28.83%)	<0.001^b^
Chronic liver disease, *n* (%)	184(8.79%)	147(8.91%)	37(8.33%)	0.701^b^
Chronic kidney disease, *n* (%)	106(5.06%)	83(5.03%)	23(5.18%)	0.900^b^
Chronic pulmonary disease, *n* (%)	615(29.38%)	474(28.74%)	141(31.76%)	0.216^b^
**Laboratory findings on the first day in ICU**				
Hb (g/l), median (IQR)	121.00(95.00,139.00)	122.00(95.50,140.00)	117.00(93.25,133.75)	0.003^*^
Creatinine (μmol/l), median (IQR)	76.80(58.10,122.80)	75.90(58.07,118.40)	80.90(58.65,135.68)	0.069^*^
Alb (g/l), median (IQR)	33.69(29.00,37.76)	33.90(29.11,37.73)	32.45(28.26,37.82)	0.133^*^
CRP (mg/l), median (IQR)	23.08(7.22,70.28)	21.53(5.89,73.14)	28.28(10.96,64.47)	0.002^*^
**Prognosis**				
Length of ICU stay (days), median (IQR)	8.00(4.00,22.00)	8.00(4.00,22.00)	12.00(6.00,24.00)	<0.001^*^
Hospital mortality, *n* (%)	208(9.94)	152(9.22)	56(12.61)	0.034^b^

a Mann Whitney U test

b Chi-square test;

**Abbreviations**: BMI, body mass index; APACHE, acute physiology and chronic health evaluation; ICU, Intensive care unit; SHR, stress hyperglycemia ratio; IQR, interquartile range.

**Table 2:** Basic characteristics of participants by SHR.

Characteristics	Overall *N* = 2093	Stress hyperglycemia ratio				*P value*
		Q1 (SHR < 0.735) *N* = 523	Q2 (0.735 ≤ SHR < 0.939) *N* = 523	Q3 (0.939 ≤ SHR < 1.243) *N* = 523	Q4 (SHR ≥ 1.243) *N* = 524	
Age (years)	66.00(54.00,77.00)	61.00(52.00,72.00)	64.00(52.00,75.00)	68.00(56.00,79.00)	70.00(57.25,80.00)	<0.001
Sex(Male)	1236(59.05%)	317(60.61)	287(54.88%)	318(60.80%)	314(59.92)	0.163
BMI (kg/m^2^)	23.90(20.35,26.80)	21.80(19.30,26.10)	22.70(19.90,26.60)	23.90(21.50,27.00)	25.40(21.80,27.20)	<0.001
Smoking, *n* (%)	548(26.18%)	143(27.34%)	139(26.58%)	130(24.86%)	136(25.95%)	0.826
Drinking, *n* (%)	322(15.38%)	85(16.25%)	78(14.91%)	80(15.30%)	79(15.08%)	0.933
Mechanical ventilation, *n* (%)	589(28.14%)	137(26.20%)	132(25.24%)	157(30.02%)	163(31.11%)	0.096
APACHEⅡpoints on the first day in ICU (points), median (IQR)	22.00(18.00,25.00)	21.00(18.00,23.00)	21.00(18.00,24.00)	22.00(19.00,25.00)	23.00(20.00,26.00)	<0.001
**Comorbidity**						
Hypertension, *n* (%)	811(38.75%)	187(35.76%)	211(40.34%)	203(38.81%)	210(40.07%)	0.403
Congestive heart disease, *n* (%)	519(24.80%)	138(26.39%)	127(24.28%)	134(25.62%)	120(22.90%)	0.576
Cerebrovascular disease, *n* (%)	476(22.74%)	102(19.50%)	108(20.65%)	122(23.33%)	144(27.48%)	0.011
Chronic liver disease, *n* (%)	184(8.79%)	36(6.88%)	43(8.22%)	46(8.80)	59(11.26%)	0.087
Chronic kidney disease, *n* (%)	106(5.06%)	20(3.82%)	29(5.54%)	28(5.35%)	29(5.54%)	0.521
Chronic pulmonary disease, *n* (%)	615(29.38%)	140(26.76%)	148(28.30%)	160(30.59%)	167(31.87%)	0.267
**Laboratory findings on the first day in ICU**						
Hb (g/l), median (IQR)	121.00(95.00,139.00)	116.00(91.00,139.00)	119.00(95.00,139.00)	124.00(99.00,140.00)	123.00(96.25,140.00)	0.032
Creatinine (μmol/l), median (IQR)	76.80(58.10,122.80)	75.10(57.00,112.40)	76.80(57.30,116.20)	78.0(59.56,129.90)	78.54(59.53,134.33)	0.130
Alb (g/l), median (IQR)	33.69(29.00,37.76)	33.00(28.40,37.20)	33.90(29.50,37.80)	33.90(28.60,37.83)	33.79(29.33,37.96)	0.084
CRP (mg/l), median (IQR)	23.08(7.22,70.28)	17.52(6.25,57.14)	20.49(6.00,69.31)	23.81(7.74,75.80)	33.28(8.94,83.28)	<0.001
**Prognosis**						
Length of ICU stay (days), median (IQR)	8.00(4.00,22.00)	8.00(4.00,22.00)	9.00(5.00,22.00)	8.00(4.00,22.00)	8.00(4.00,22.00)	0.463
Hospital mortality, *n* (%)	208(9.94)	49(9.37)	52(9.94)	51(9.75)	56(10.69)	0.910
Delirium, *n* (%)	444(21.21)	71(13.58)	101(19.31)	127(24.28)	145(27.72)	<0.001

a Kruskal Wallis test

b Chi-square test;

**Abbreviations**: BMI, body mass index; APACHE, Acute physiology and chronic health evaluation; ICU, Intensive care unit; IQR, interquartile range.

### *Relationship between SHR* and delirium

Logistic regression models analyzing the association between SHR and delirium episodes, in which SHR levels were significantly and positively associated with delirium episodes in all three models. In the fully adjusted model, the risk of delirium episodes increased by 85.0% (OR: 1.85, 95% CI: 1.46–2.36) for each unit increase in the patient’s SHR level. We also performed multivariate logistic regression analyses of quartiles of SHR levels at the occurrence of delirium in ICU patients. In the fully adjusted model, participants in the highest SHR quartile had a significantly higher risk of delirium episodes while in the ICU compared to participants in the lowest SHR quartile (OR: 1.88, 95% CI:1.35–2.62, Table 3).

**Table 3:** Relationship between SHR and delirium in ICU patients.

SHR	*OR(95%CI), P value*		
	Crude model (Model 1)	Minimally adjusted model (Model 2)	Fully adjusted model(Model 3)
Continuous	2.08(1.65-2.61), < 0.001	1.83(1.44-231), < 0.001	1.85(1.46-2.36), < 0.001
**SHR classification**			
Quartile 1	Reference	Reference	Reference
Quartile 2	1.52(1.09-2.12), 0.013	1.44(1.03-2.01), 0.032	1.40(0.99-1.97), 0.055
Quartile 3	2.04(1.48-2.81), < 0.001	1.79(1.29-2.48), < 0.001	1.76(1.26-2.46), < 0.001
Quartile 4	2.44(1.78-3.34), < 0.001	2.02(1.47-2.79), < 0.001	1.88(1.35-2.62), < 0.001
*P* for trend	<0.001	<0.001	0.001

Model 1: No covariates were adjusted.

Model 2: Age and BMI were adjusted.

Model 3: Age, BMI, cerebrovascular diseases, hemoglobin, C-reactive protein, APACHE Ⅱ points on the first day in ICU, length of ICU stay, and hospital mortality were adjusted.

Abbreviations: SHR: stress hyperglycemia ratio.

**Fig 2:**
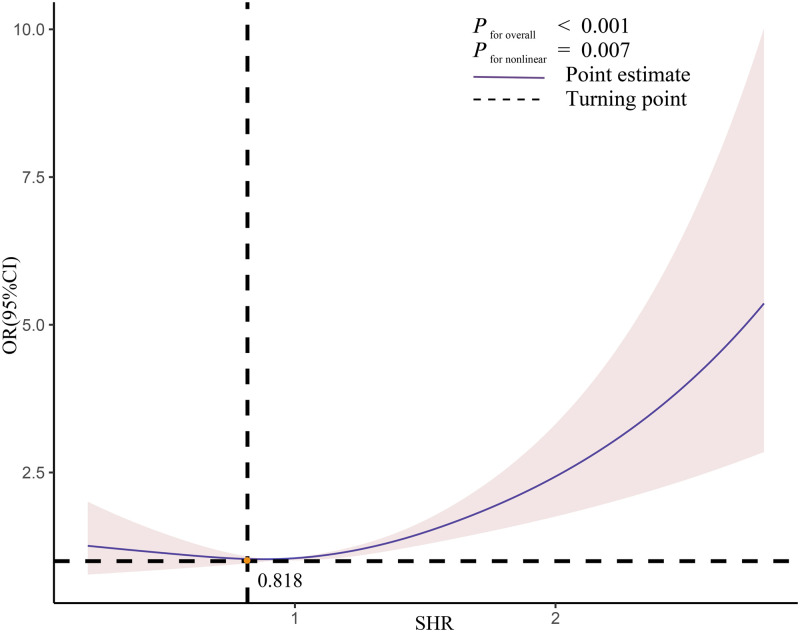
The relationship between SHR and delirium. The solid red line represents the smoothing curve fit between variables. Blue bands represent the 95% confidence interval from the fit.

The RCS analysis revealed a J-shaped nonlinear relationship between SHR and delirium risks. Initially, the risk of delirium decreased as SHR increased. However, once the SHR exceeded 0.818 these risks began to escalate significantly (Fig 2). Moreover, in the threshold analysis, for every one increase in SHR, participants with SHR less than 0.818 had a 77.7% reduction in the incidence of delirium (OR=0.78; 95% CI: 0.61–0.99), SHR 0.818 or above had a 50.2% increased incidence of delirium in participants (OR=1.50; 95% CI: 1.20–1.89, Table 4).

**Table 4:** Threshold effects of SHR on odds of delirium prevalence were analyzed using two piece linear regression models.

Delirium	OR (95%CI)	*P* value
Turning point	0.818	
SHR < 0.818	0.777(0.611-0.989)	0.040
SHR >=0.818	1.502 (1.196-1.886)	0.040

**Abbreviation:** Age, BMI, cerebrovascular diseases, hemoglobin, C-reactive protein, APACHEⅡpoints on the first day in ICU, length of ICU stay, and hospital mortality were adjusted. SHR, stress hyperglycemia ratio; OR: odds ratio; 95% CI: 95% confidence interval.

### Subgroup and sensitivity analysis

**Fig 3:**
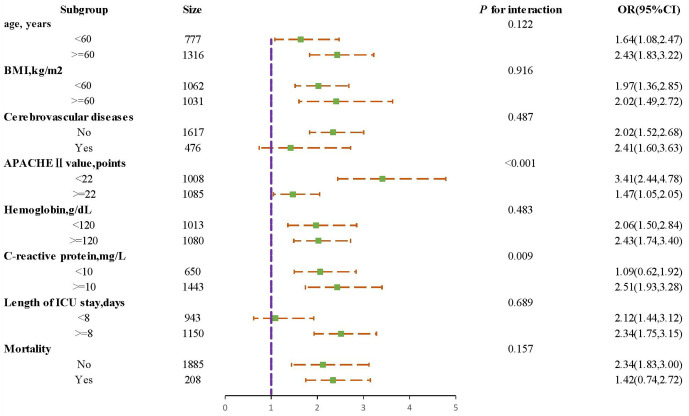
Subgroup analysis for the association between SHR and delirium. The analysis was adjusted for age, BMI, diabetes, hemoglobin, CRP, APACHE Ⅱ points on the first day in ICU, length of ICU stay, and mortality **Abbreviations**: BMI, body mass index; APACHE, acute physiology and chronic health evaluation; SHR, stress hyperglycemia ratio.

We selected the demographic key variables of age, BMI, diabetes, hemoglobin, CRP, APACHE Ⅱ points on the first day in ICU, length of ICU stay, and mortality to conduct subgroup analyses and tests of interaction outcomes, validating robustness across groups (Fig 3).

## Discussion

In this study, a positive correlation was identified between SHR and the occurrence of delirium in ICU patients. This correlation was sustained across all three models, thereby suggesting that increasing values of SHR are associated with an increased risk of delirium in ICU patients. When evaluating the dose,response relationship between SHR and delirium in critically ill patients, we found an inflection point where there was a significant positive correlation between SHR and delirium when SHR more than 0.818. These findings highlight that abnormalities in glucose metabolism may play a key role in the pathogenesis of delirium and the potential for SHR to serve as a target for early intervention for delirium in ICU patients. It is noteworthy that the APACHE II score and C-reactive protein on admission were identified as significant modifiers of this association, emphasizing the necessity for an approach that addresses different disease severity and inflammatory indicators in the assessment and management of delirium in critically ill patients.

SHR has been extensively utilized as a biomarker for the assessment of glucose metabolism dysfunction and disease severity in critically ill patients, facilitating prognostication and monitoring of diseases such as metabolic syndrome [20,21]. It has been shown that there is an important correlation between SHR and central nervous system health [22]. A cohort study in mainland China documented that both lower and higher SHR were associated with an increased risk of delirium in elderly hospitalized patients, and that admission SHR may serve as a promising predictor of delirium [13]. A meta-analysis reported that SHR serves as a significant predictor of stroke prognosis, with a 2.84-fold increased risk of stroke recurrence for each unit of increase, and that neurological deficits were associated with a 2.84-fold increased risk of stroke recurrence for each unit of increase in SHR unit was associated with a 2.84-fold increase in the risk of stroke recurrence and a 1.73-fold increase in the risk of neurologic deficit [23]. These findings further confirmed the existence of a correlation between the SHR ratio and diseases of the central nervous system. Delirium, an acute syndrome of the central nervous system, is also more common in critically ill patients. To our knowledge, this is the first study to examine the relationship between SHR and the risk of delirium in ICU patients. According to our findings, the risk of delirium increases by 76.0% for each unit increase in SHR. These results indicate that heightened attention should be given to the risk of delirium when the SHR index is elevated in critically ill patients, and that proactive interventions should be implemented to prevent delirium episodes based on changes in the SHR ratio. In addition, a systematic evaluation of potential modifiable risk factors for long-term cognitive impairment after critical illness found that hypoglycemia, hyperglycemia, fluctuations in serum glucose levels, and in-hospital acute stress a correlation between symptoms and cognitive dysfunction in critically ill patients [24]; however, this association was weak and inconsistent. This result may be due to differences in the included literature, and future studies should be conducted extensively to explore the relationship between SHR and acute cognitive dysfunction in critically ill patients to provide the literature base for meta-analyses. Similarly, a prospective cohort study in the Netherlands involving 2,745 critically ill patients found that the occurrence of hyperglycemia, as well as the simultaneous occurrence of hyperglycemia and hypoglycemia on the same day, was associated with a shift to delirium in nondiabetic patients [25]. All of the above studies have highlighted that controlling blood glucose levels can help to reduce the risk of acute cognitive alterations in critically ill patients, thus validating our results and highlighting the potential clinical value of SHR in controlling delirium in critically ill patients.

Several potential mechanisms could explain the observed association. Firstly, hyperglycaemic states increase the difference between intra- and extracellular blood glucose concentrations, leading to abnormal intracellular osmotic pressure, which affects intracellular energy metabolism, particularly in brain cells, which may suffer from a lack of energy supply, leading to impaired brain function [26]. Secondly, hyperglycaemia also activates protein kinase C and NADPH oxidase, increases reactive oxygen species (ROS) levels and decreases nitric oxide synthase, leading to reduced reperfusion and exacerbating more neuronal damage [27,28]. Thirdly, the body may experience inflammatory responses and oxidative stress in a hyperglycaemic environment, leading to mitochondrial dysfunction and endothelial dysfunction, which can affect blood flow and neurological function in the brain through a number of pathways, leading to cognitive dysfunction [29–31]. Finally, hyperglycaemia may affect the synthesis and release of neurotransmitters, such as elevated levels of excitatory neurotransmitters such as glutamate, which may cause hyperexcitability of neurons, leading to cognitive impairment and delirium [14,32,33]. This also suggests that SHR is a therapeutic target to reduce the risk of delirium.

Notably, the J-shaped nonlinear relationship between SHR and delirium was moderated by the APACHE II score on admission and C-reactive protein. The APACHE II score is a commonly used condition assessment system to evaluate the severity and prognosis of critically ill patients. Evidence suggests that the more severe the condition of critically ill patients, the more severe the risk of glucose metabolic disorders and delirium, which in turn aggravate the severity of the disease, interacting with each other and influencing each other [34,35]. Furthermore, C-reactive protein is a widely accepted clinical indicator of the body’s inflammatory response. A retrospective study found that the SHR ratio interacts with inflammatory markers, and both together influence the risk of delirium in elderly patients with community-acquired pneumonia [36], which is consistent with the results of the present study and emphasizes the importance of considering the role of inflammatory substances while evaluating delirium in critically ill patients according to the SHR index.

Our study has several strengths, including the fact that this study was conducted in a general hospital in a large city, as well as the fact that the study adjusted for potential con-founders and performed subgroup analyses to test the robustness of the relationship between SHR levels and the risk of delirium in different populations. However this study still has some limitations. First, this study was a retrospective, single-center investigation, and we were unable to respond to the time,series temporal association between SHR and delirium. More longitudinal prospective studies are needed to validate the temporal relationship. Second, despite adjusting for multiple known con-founders, we still could not rule out the influence of all potential con-founders. Third, because the SHR was measured only at one point in time, we were unable to account for changes in the SHR over time, which may have affected delirium incidence. Fourth, the study failed to collect data on participants receiving hypoglycemic therapy and using medications that affect glucose concentrations, thus preventing assessment of any other correlations between SHR and hypoglycemic therapy.

## Conclusion

This study demonstrates a J-shaped nonlinear relationship between SHR and the occurrence of delirium in critically ill patients. This correlation was influenced by the interaction of APACHE II score and C-reactive protein at admission. However, further prospective studies are needed to confirm our findings.

## Supporting information

S1 Fig Flowchart of the study. (PDF)

S2 Fig Frequency distribution of SHR of study participants. (PDF)

S1 Table Collinearity diagnostics steps. (DOCX)

S2 Table Spearman’s correlation between SHR levels and the risk factors associated with delirium, hospital mortality, length of ICU stay. (DOCX)
