# Supplementary material for: J-shaped relationship between stress hyperglycemia ratio and delirium risk in critically ill patients: A population-based study
Source: PLoS One. 2026 Jun 5;21(6):e0350652. doi: 10.1371/journal.pone.0350652 (PMC13240923; doi:10.1371/journal.pone.0350652)
Supplement: S1 Fig — (PDF) [file pone.0350652.s001.pdf]

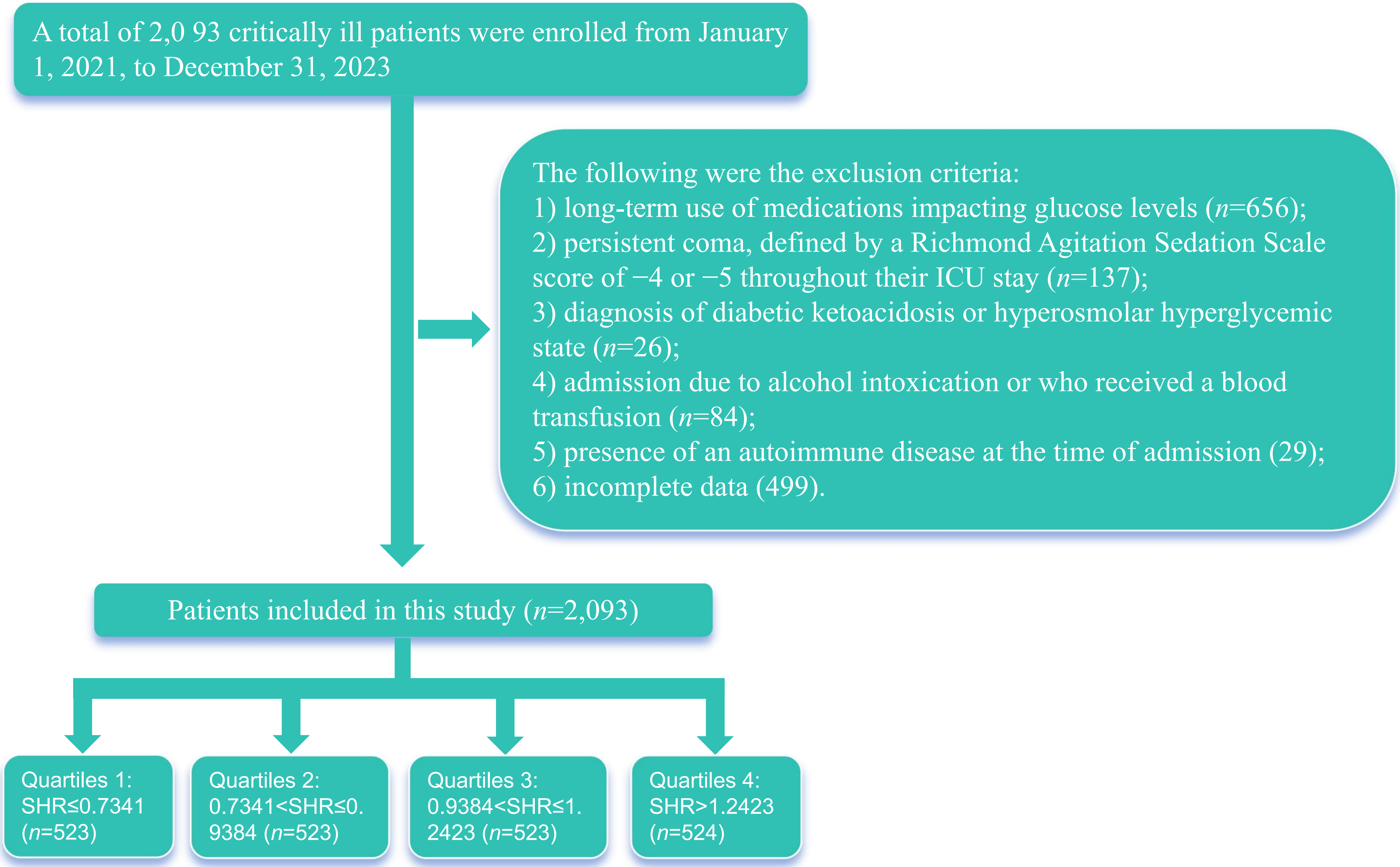
A total of 2,0 93 critically ill patients were enrolled from January 1, 2021, to December 31, 2023
The following were the exclusion criteria:
1) long-term use of medications impacting glucose levels (n=656);
2) persistent coma, defined by a Richmond Agitation Sedation Scale score of −4 or −5 throughout their ICU stay (n=137);
3) diagnosis of diabetic ketoacidosis or hyperosmolar hyperglycemic state (n=26);
4) admission due to alcohol intoxication or who received a blood transfusion (n=84);
5) presence of an autoimmune disease at the time of admission (29);
6) incomplete data (499).
Patients included in this study (n=2,093)
Quartiles 1: SHR≤0.7341 (n=523)
Quartiles 2: 0.7341<SHR≤0. 9384 (n=523)
Quartiles 3: 0.9384<SHR≤1. 2423 (n=523)
Quartiles 4: SHR>1.2423 (n=524)
