# Supplementary figures and images for: J-shaped relationship between stress hyperglycemia ratio and delirium risk in critically ill patients: A population-based study
Source: PLoS One. 2026 Jun 5;21(6):e0350652. doi: 10.1371/journal.pone.0350652 (PMC13240923; doi:10.1371/journal.pone.0350652)

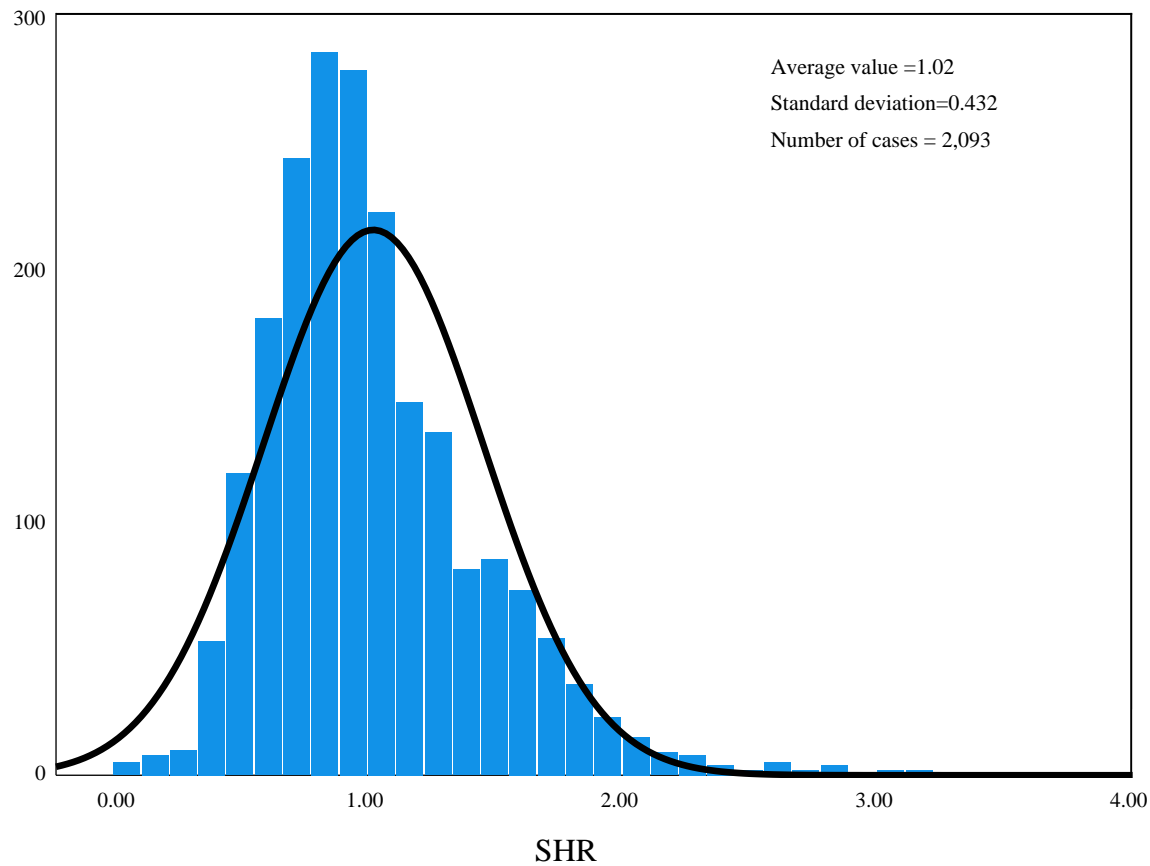

Supplement: S2 Fig — (PDF) [file pone.0350652.s002.pdf]
