# Supplementary material for: J-shaped relationship between stress hyperglycemia ratio and delirium risk in critically ill patients: A population-based study
Source: PLoS One. 2026 Jun 5;21(6):e0350652. doi: 10.1371/journal.pone.0350652 (PMC13240923; doi:10.1371/journal.pone.0350652)
Supplement: S1 Table — (DOCX) [file pone.0350652.s003.docx]

**Table S1. Collinearity diagnostics steps.**

| *Variable* | *β* | *SE* | *P* | *Tolerance* | *VIF* |
| --- | --- | --- | --- | --- | --- |
| Sex | -0.013 | 0.018 | 0.546 | 0.922 | 1.084 |
| Age | 0.095 | 0.001 | <0.001 | 0.886 | 1.128 |
| BMI | 0.126 | 0.002 | <0.001 | 0.990 | 1.011 |
| Smoking | -0.034 | 0.020 | 0.118 | 0.972 | 1.029 |
| Drinking | -0.023 | 0.024 | 0.275 | 0.967 | 1.034 |
| Hypertension | -0.024 | 0.019 | 0.282 | 0.870 | 1.150 |
| Congestive heart failure | 0.007 | 0.022 | 0.773 | 0.865 | 1.156 |
| Cerebrovascular diseases | 0.029 | 0.025 | 0.191 | 0.934 | 1.071 |
| Chronic liver disease | -0.011 | 0.031 | 0.611 | 0.987 | 1.013 |
| Chronic kidney disease | -0.009 | 0.040 | 0.679 | 0.954 | 1.048 |
| Chronic pulmonary disease | -0.013 | 0.036 | 0.754 | 0.272 | 3.673 |
| Mechanical ventilation | 0.051 | 0.037 | 0.216 | 0.267 | 3.741 |
| APACHEⅡ points on the first day in ICU | 0.095 | 0.002 | <0.001 | 0.982 | 1.018 |
| Hb | -0.075 | <0.001 | 0.001 | 0.834 | 1.199 |
| Creatinine | 0.002 | <0.001 | 0.930 | 0.902 | 1.108 |
| Alb | 0.029 | 0.001 | 0.201 | 0.866 | 1.154 |
| CRP | -0.021 | <0.001 | 0.333 | 0.939 | 1.065 |
| Length of ICU stay | 0.164 | 0.001 | <0.001 | 0.977 | 1.023 |
| Hospital mortality | 0.027 | 0.030 | 0.220 | 0.947 | 1.056 |

VIF = 1/(1-R^2^). VIF step-by-step screening method: Calculate the VIF of each variable. If the maximum VIF value ≥ 5, remove the variable with the maximum VIF value.

Abbreviations: VIF, variance inflation factor. Other abbreviations as presented in Table 1.
