# Supplementary material for: J-shaped relationship between stress hyperglycemia ratio and delirium risk in critically ill patients: A population-based study
Source: PLoS One. 2026 Jun 5;21(6):e0350652. doi: 10.1371/journal.pone.0350652 (PMC13240923; doi:10.1371/journal.pone.0350652)
Supplement: S2 Table — (DOCX) [file pone.0350652.s004.docx]

**Table S2.** **Spearman's correlation between SHR levels and the risk factors associated with delirium, hospital mortality, length of ICU stay.**

| Variable | | Delirium | Hospital mortality | Length of ICU stay | SHR |
| --- | --- | --- | --- | --- | --- |
| Delirium | Pearson correlation | - | - | - | - |
|  | *P* | - | - | - | - |
| Hospital mortality | Pearson correlation | 0.046^*^ | - | - | - |
|  | *P* | 0.034 | -- | - | - |
| Length of ICU stay | Pearson correlation | 0.157^**^ | 0.006 | - | - |
|  | *P* | <0.001 | 0.785 | - | - |
| SHR | Pearson correlation | 0.152^**^ | 0.014 | 0.019 | - |
|  | *P* | <0.001 | 0.528 | 0.395 | - |

*<0.05;**<0.01. Abbreviations: Abbreviations as presented in Table 1.
